# Association of genetic variants previously implicated in coronary artery disease with age at onset of coronary artery disease requiring revascularizations

**DOI:** 10.1371/journal.pone.0211690

**Published:** 2019-02-06

**Authors:** Charlotte Andersson, Maria Lukács Krogager, Regitze Kuhr Skals, Emil Vincent Rosenbaum Appel, Christian Theil Have, Niels Grarup, Oluf Pedersen, Jørgen L. Jeppesen, Ole Dyg Pedersen, Helena Dominguez, Ulrik Dixen, Thomas Engstrøm, Niels Tønder, Dan M. Roden, Steen Stender, Gunnar H. Gislason, Henrik Enghusen-Poulsen, Torben Hansen, Lars Køber, Christian Torp-Pedersen, Peter E. Weeke

**Affiliations:** 1 Department of Cardiology, Herlev and Gentofte Hospital, Hellerup, Denmark; 2 Unit of Epidemiology and biostatistics, Aalborg University Hospital, Aalborg, Denmark; 3 Department of Cardiology, Aalborg University Hospital, Aalborg, Denmark; 4 The Novo Nordisk Foundation Center for Basic Metabolic Research, Section of Metabolic Genetics, Faculty of Health and Medical Sciences, University of Copenhagen, Copenhagen, Denmark; 5 Department of Internal Medicine, Section of Cardiology, Amager and Hvidovre Hospital Glostrup, Glostrup, Denmark; 6 Department of Cardiology, Roskilde Hospital, Roskilde, Denmark; 7 Department of Cardiology, Bispebjerg and Frederiksberg Hospital, Bispebjerg, Denmark; 8 Department of Biomedicine, University of Copenhagen, Copenhagen, Denmark; 9 Department of Cardiology, Amager and Hvidovre Hospital Hvidovre, Hvidovre, Denmark; 10 Department of Cardiology, Copenhagen University Hospital Rigshospitalet, Copenhagen, Denmark; 11 Department of Cardiology, Nephrology, and Endocrinology, Hillerød Hospital, Hillerød, Denmark; 12 Departments of Medicine, Pharmacology, and Biomedical Informatics, Vanderbilt University Medical Center, Nashville, Tennessee, USA; 13 Department of Clinical Biochemistry, Copenhagen University Hospital, Gentofte, Denmark; 14 The Danish Heart Foundation, Copenhagen, Denmark; 15 The National Institute of Public Health, University of Southern Denmark, Copenhagen, Denmark; 16 Laboratory of Clinical Pharmacology, Rigshospitalet, University of Copenhagen, Copenhagen, Denmark; 17 Department of Clinical Pharmacology, Bispebjerg and Frederiksberg Hospital, University of Copenhagen, Copenhagen, Denmark; 18 Department of Clinical Medicine, Faculty of Health and Medical Sciences, University of Copenhagen, Copenhagen, Denmark; 19 Steno Diabetes Center, Gentofte, Denmark; 20 Department of Health Science and Technology, Aalborg University Hospital, Aalborg, Denmark; 21 Department of Medicine, Vanderbilt University Medical Center, Nashville, Tennessee, United States of America; China Medical University, TAIWAN

## Abstract

**Background:**

The relation between burden of risk factors, familial coronary artery disease (CAD), and known genetic variants underlying CAD and low-density lipoprotein cholesterol (LDL-C) levels is not well-explored in clinical samples. We aimed to investigate the association of these measures with age at onset of CAD requiring revascularizations in a clinical sample of patients undergoing first-time coronary angiography.

**Methods:**

1599 individuals (mean age 64 years [min-max 29–96 years], 28% women) were genotyped (from blood drawn as part of usual clinical care) in the Copenhagen area (2010–2014). The burden of common genetic variants was measured as aggregated genetic risk scores (GRS) of single nucleotide polymorphisms (SNPs) discovered in genome-wide association studies.

**Results:**

Self-reported familial CAD (prevalent in 41% of the sample) was associated with -3.2 years (95% confidence interval -4.5, -2.2, p<0.0001) earlier need of revascularization in sex-adjusted models. Patients with and without familial CAD had similar mean values of CAD-GRS (unweighted scores 68.4 vs. 68.0, p = 0.10, weighted scores 67.7 vs. 67.5, p = 0.49) and LDL-C-GRS (unweighted scores 58.5 vs. 58.3, p = 0.34, weighted scores 63.3 vs. 61.1, p = 0.41). The correlation between the CAD-GRS and LDL-C-GRS was low (r = 0.14, p<0.001). In multivariable adjusted regression models, each 1 standard deviation higher values of LDL-C-GRS and CAD-GRS were associated with -0.70 years (95% confidence interval -1.25, -0.14, p = 0.014) and -0.51 years (-1.07, 0.04, p = 0.07) earlier need for revascularization, respectively.

**Conclusions:**

Young individuals presenting with CAD requiring surgical interventions had a higher genetic burden of SNPs relating to LDL-C and CAD (although the latter was statistically non-significant), compared with older individuals. However, the absolute difference was modest, suggesting that genetic screening can currently not be used as an effective prediction tool of when in life a person will develop CAD. Whether undiscovered genetic variants can still explain a “missing heritability” in early-onset CAD warrants more research.

## Introduction

Coronary artery disease (CAD) begins as a subclinical process, generally spanning several decades before eventually becoming symptomatic. There is, however, a substantial individual variability in age at onset of symptoms and approximately 10% of myocardial infarctions present in young adults (i.e., as premature disease, generally defined as onset <55 years and <65 years in men and women, respectively).[1] Because the coronary atherosclerotic burden is a product of the numbers of risk factors multiplied by the time exposed to them, young individuals affected by overt disease would be expected to have a higher burden of risk factors than individuals that are older at age of onset. Whereas modifiable cardiovascular risk factors have been reported to account for the majority of variation in risk of CAD even in young individuals,[2] a familial history of CAD appears also to be a very strong risk factor for disease in these patients.[3–5] Such relation could suggest a significant contribution of genetics in the pathogenesis of CAD in young individuals. Yet, familial CAD reflects both genetic and non-genetic risk factors (including e.g. smoking, physical inactivity, and adverse dietary habits) and the importance of known genetic variants for age at onset of disease is not well-explored. Although the isolated importance of genetic predisposition for CAD has been suggested to be greater in young compared with older individuals in the general population,[6] there appears to be an important risk modification by a healthy behavior, suggesting that genetic variants only have a modest influence on CAD risk if all other risk factors are ideal.[7]

In this work, we aimed to study the distribution of traditional risk factors and genetic risk variants of CAD and low-density lipoprotein cholesterol (LDL-C) levels in young vs. older patients undergoing first-time coronary revascularization therapy to further our understanding about the role of known genetic variants associated with CAD and LDL-C levels, respectively, in premature CAD.

## Methods

The data, analytic methods, and study materials can be made available to other researchers for purposes of reproducing the results or replicating the procedure upon request (through collaboration). The Copenhagen Cardiovascular Genetic study (COGEN) is a biobank that has collected superfluous whole blood from patients admitted to six cardiology departments in the Capital region of Copenhagen from 2010–2017. COGEN currently contains samples from ~80,000 individuals. Since all permanent residents hold a Danish identification number, these data can be linked with various clinical databases at an individual level. To date, a sample of ~5200 individuals from COGEN who have had at least one coronary angiogram performed between 2010–2014 have been genotyped as described below and in the online supplemental.

Data on the angiograms were collected from the Eastern Danish Heart Registry, which is a clinical database, where information on demographic (age, sex), risk factors and comorbidities (e.g., smoking status, diabetes, known high blood cholesterol levels, left ventricular ejection fraction, significant valve disease, hypertension, and self-reported history of familial coronary artery disease), and coronary pathology has been routinely entered on all patients in the Eastern region of Denmark.[8] Information on the type of clinical presentation is also available (ST-elevation myocardial infarction [STEMI], non-ST-elevation myocardial infarction [NSTEMI], unstable angina pectoris [UAP], and stabile angina pectoris [SAP]).[9]

### Study population

For the current investigation, we included those patients who had no prior known CAD and who were found to have significant CAD requiring interventions by angioplasty or coronary artery bypass surgery. We defined premature cardiovascular disease as a first-event occurring before age 55 years in men and 65 years in women, in agreement with prior studies.[10] In addition, we defined very young patients as men being younger than 35 years and women below age 45 years. We also divided non-premature coronary artery disease into two groups (intermediate age >55–70 years in men and >65–80 years in women, and old age >70 years for men and >80 years for women). These additional subdivisions were based on arbitrary cutoff points.

### Genotyping and derivation of polymorphic genetic risk scores

Genome-wide genotyping was performed using the Illumina Infinium Human CoreExome BeadChip (Illumina, San Diego, CA, USA) and updated to build 37 (i.e. hg19). We employed a standard quality control (QC) pipeline after which a total of 5128 individuals and 539004 SNPs were available for further analyses (of which the majority were not first-time interventions). We then imputed the data on the Sanger Imputation Server to the first release reference panel of human haplotypes by the Haplotype Reference Consortium (**HRC** version r1.1).[11] See **S1 File** for detailed information on QC and imputation.

Based on data from prior genome-wide association studies of individuals of European ancestry, we compiled additive and weighted genetic risk scores (GRS) for CAD and LDL-C. Overall, 67 and 58 independent single nucleotide polymorphisms (SNPs) previously identified from genome-wide association studies were included for the CAD and LDL-C risk scores, respectively. Full SNP lists are available in **S1 and S2 Tables**. We constructed 2 different risk scores, by orienting the SNPs such that the risk *increasing* allele was always coded as the alternative allele and the risk *decreasing* allele as the reference allele, regardless of allele frequency. In the unweighted GRS (uGRS), we assigned 1 point per risk increasing allele assuming an additive risk model. GRS_i_ is the unweighted GRS for individual *i*, s_ij_ is the number of effect increasing alleles for the *j*’th SNP for individual *i* and N is the number of SNPs in the GRS:

$$u{GRS}_{i}=\sum_{j=1}^{N}s_{ij}.$$

In the weighted GRS (wGRS), the risk alleles were weighted according to beta-estimates derived from the prior genome-wide association studies.[12] Thus, for the wGRS, we multiplied each allele with the reported effect size, after which we summed up the total value of all weighted SNPs. We then normalized by dividing by the average effect size. wGRS_i_ is the weighted GRS for individual *i*, s_ij_ is the number of effect increasing alleles for the *j*’th SNP for individual *i*, N is the number of SNPs in the GRS and β_j_ is the reported effect size for the *j*’th SNP.

$${wGRS}_{i}=\frac{N}{\sum_{j=1}^{N}\beta_{j}}\sum_{j=1}^{N}\beta_{j}s_{ij}.$$

Of the CAD related SNPs, 51 were directly genotyped and 16 additional SNPs were available through statistical imputation, of which 3 SNPs were proxy variants (r^2^>0.8), **S1 Table**. Similar, for low-density lipoprotein cholesterol (LDL-C),[13, 14] 43 SNPs were directly genotyped and additionally 11 SNPs were available through statistical imputation, of which 3 SNPs were proxy variants (r^2^>0.8), **S2 Table**. Eligible proxy variants were identified using https://analysistools.nci.nih.gov/LDlink/.

### Statistics

Characteristics of the overall sample and various age-groups are presented as mean values ± standard deviation (SD) for continuous variables and as number (percentage) for discrete variables. Tests for trends across the four age-groups were performed by the Cochran-Armitage trend test for discrete variables and by general linear models (with age-group as a continuous, independent variable) for continuous variables.

**Fig 1:**
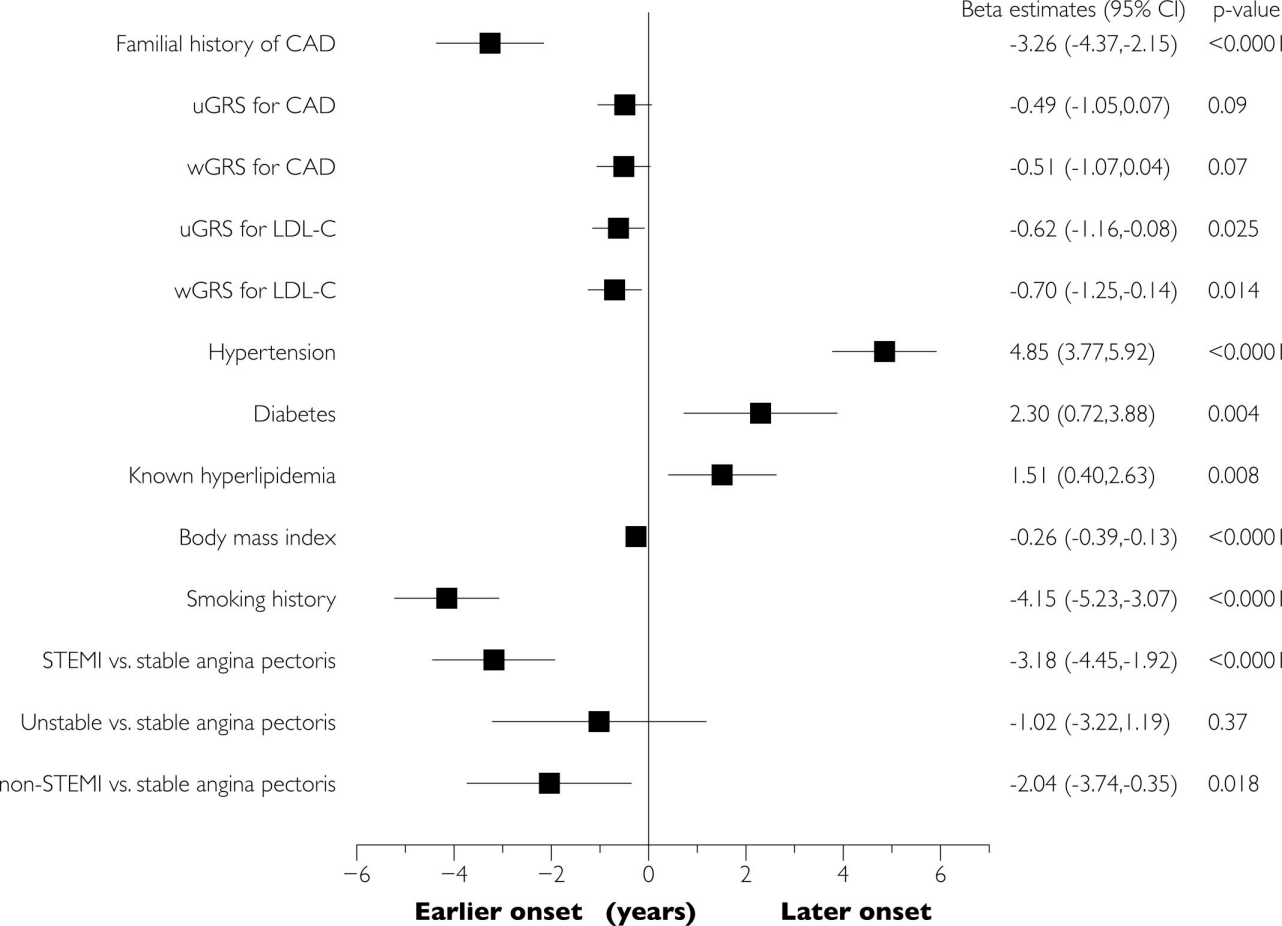
Sex-adjusted analyses of the association of various factors with age at onset of obstructive CAD. Parameter estimates (i.e., years below/above the overall mean age of onset of CAD) from the linear regression analysis, adjusted for sex. wGRS = weighted genetic risk score. uGRS = unweighted genetic risk score. Models of genetic risk scores were additionally adjusted for genetic principal components 1 and 2.

**Fig 2:**
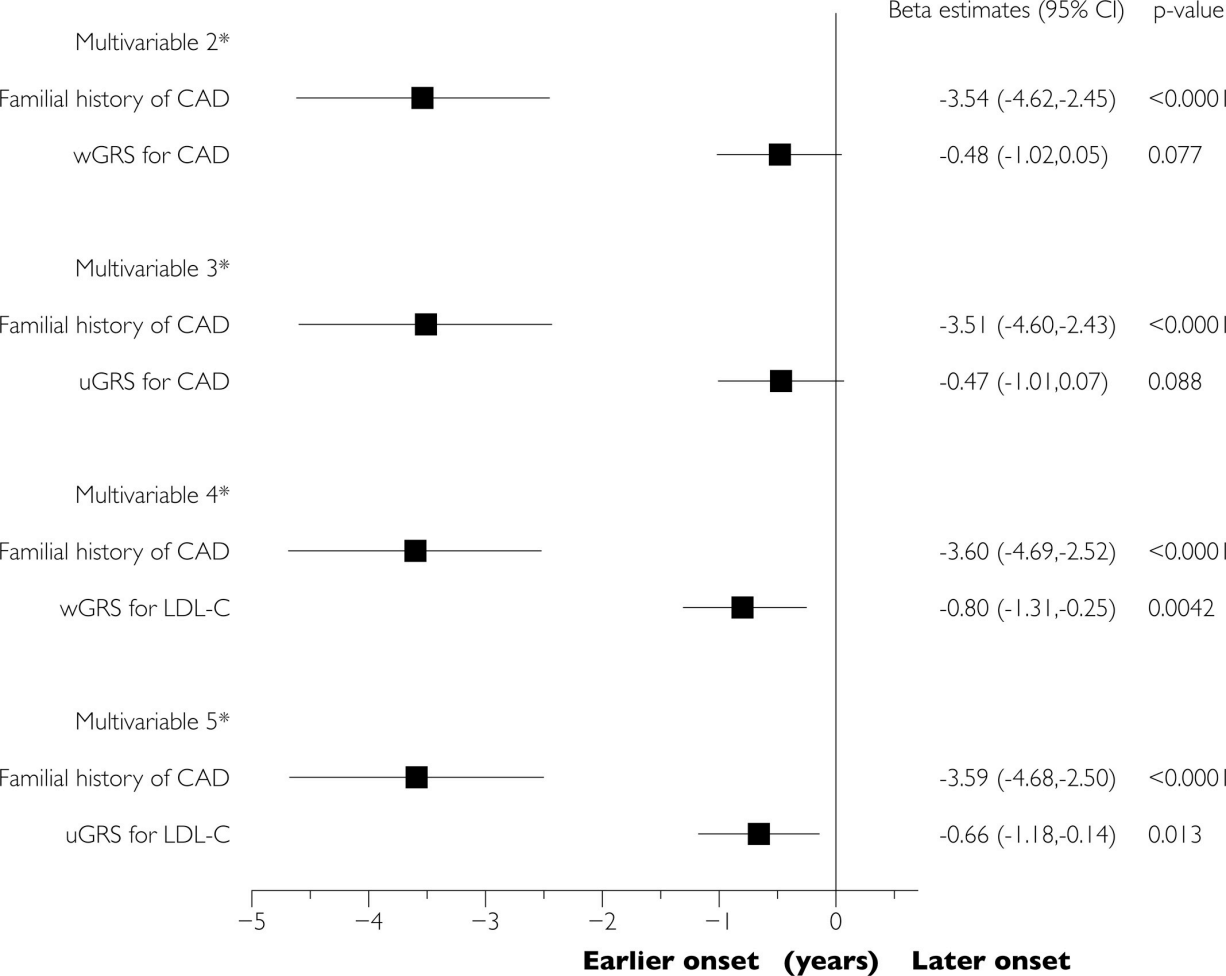
Multivariable-adjusted analyses of the association of various factors with age at onset of obstructive CAD. Parameter estimates (i.e., years below/above the overall mean age of onset of CAD) from the linear regression analysis. * Model 2–5 were adjusted for sex, principal components 1 and 2, familial CAD, smoking, body mass index, diabetes, hypertension, and known high blood cholesterol prior to revascularization. Model 2 analyzed wGRS for CAD, model 3 uGRS for CAD, model 4 wGRS for LDL-C, and model 5 uGRS for LDL-C. wGRS = weighted genetic risk score.

We estimated the association of GRS with age at onset of disease by linear regression models, where age at onset (as a continuous variable) was included as the dependent variable and GRS, principal components 1 and 2, and sex as the independent variables. To facilitate the comparison, GRS were standardized to mean 0 and SD 1 before analyses. We also estimated the association of the other variables with age at onset by similar univariable (**Fig 1**) and multivariable (adjusting for body mass index, known hyperlipidemia, smoking, diabetes, and hypertension; **Fig 2**) linear regression models. A two-sided p-value <0.05 was considered as statistically significant. All analyses were performed in SAS version 9.3 (SAS institute, Cary, NC, USA).

### Ethics

All data were de-identified prior to analyses. The ethics committee of Region North Jutland (N-20140048) approved the project and COGEN has permission from the Data Protection Agency (00916 GEH-2010-001).

## Results

We identified a total of 1,599 patients of Danish ancestry (genetically determined) undergoing first-time revascularization with available genotyping. The mean age was 64 years (min-max 29–96 years) and 28% were women. **Tables 1 and 2** show the full characteristics of the sample for men and women, respectively. The proportion of patients who had familial CAD, were more frequently smokers, and the mean values of LDL-C-GRS and CAD-GRS were higher in young vs. older among both men and women, **Tables 1 and 2**. However, for the youngest women (<50 years) GRS values appeared less elevated compared with men and older women. Except for the oldest ages, the proportion with known high cholesterol levels increased with advancing age, whereas the burden of LDL-C-GRS decreased. Diabetes and hypertension prevalence and body mass index values were also higher with older ages.

**Table 1:** Characteristics stratified by age for men.

	Men				P for trend
	Very young	Young	Intermediate	Old	
Cutoff values used (years)	≤40	>40–55	>55–70	>70	
N	10	297	537	308	
Age at revascularization, mean (±SD)	36.9 (2.7)	49.8 (3.6)	63.3 (4.3)	76.6 (4.6)	
BMI, mean (±SD) (kg/m^2^)	26.8 (2.9)	27.9 (4.3)	27.7 (4.2)	26.7 (3.7)	0.0003
Smoking (%)	7 (70)	176 (59.3)	299 (55.7)	125 (40.6)	<0.001
Diabetes (%)	0	28 (9.4)	81 (15.1)	50 (16.2)	0.0083
Hypertension (%)	0	102 (34.3)	272 (50.7)	175 (56.8)	<0.001
Known high cholesterol (%)	2 (20)	114 (38.4)	252 (46.9)	137 (44.5)	0.032
Familial CAD (%)	5 (50)	144 (48.5)	216 (40.2)	83 (27.0)	<0.001
Unweighted CAD-GRS, mean (±SD)	69.7 (3.6)	68.4 (4.7)	68.4 (4.7)	67.5 (4.5)	0.026
Weighted CAD-GRS, mean (±SD)	68.9 (3.9)	67.8 (4.3)	67.8 (4.3)	66.9 (4.3)	0.0067
Unweighted LDL-C-GRS (±SD)	59.2 (3.3)	59.0 (4.6)	58.4 (4.5)	58.2 (4.5)	0.024
Weighted LDL-C-GRS (±SD)	63.0 (3.6)	61.9 (5.2)	61.1 (5.1)	60.7 (5.0)	0.001
ST elevation myocardial infarction	8 (80)	179 (60.3)	267 (49.7)	128 (41.6)	<0.001
Non-ST segment myocardial infarction	1 (1)	33 (11.1)	68 (12.7)	32 (10.4)	0.58
Unstable angina pectoris	0	14 (4.7)	36 (6.7)	24 (7.8)	0.1359
Stable angina pectoris	1 (10)	71 (23.9)	166 (30.9)	124 (40.3)	<0.001

BMI, body mass index; CAD-GRS, genetic risk score for coronary artery disease; LDL-C-GRS, genetic risk score for low-density lipoprotein cholesterol.

**Table 2:** Characteristics stratified by age for women.

	Women				P for trend
	Very young	Young	Intermediate	Old	
Cutoff values used (years)	≤50	>50–65	>65–80	>80	
N	36	153	188	70	
Age at revascularization, mean (±SD)	45.5 (4.2)	58.3 (4.4)	72.7 (3.9)	85.1 (3.8)	
BMI, mean (±SD)	25.0 (4.5)	26.3 (5.1)	26.2 (4.5)	24.2 (3.8)	0.069
Smoking (%)	26 (72)	99 (64.7)	80 (42.6)	26 (37.1)	<0.001
Diabetes (%)	6 (16.7)	13 (8.5)	37 (19.7)	9 (12.9)	0.24
Hypertension (%)	8 (22.2)	67 (43.8)	125 (66.5)	49 (70.0)	<0.001
Known high cholesterol (%)	7 (19.4)	68 (44.4)	109 (58.0)	24 (34.3)	0.10
Familial CAD (%)	15 (41.7)	80 (52.3)	93 (49.5)	25 (35.7)	0.029
Unweighted CAD-GRS, mean (±SD)	66.7 (4.6)	68.5 (5.2)	68.0 (4.9)	68.1 (4.7)	0.88
Weighted CAD-GRS, mean (±SD)	66.3 (4.1)	67.9 (4.9)	67.7 (4.5)	67.4 (4.3)	0.61
Unweighted LDL-C-GRS (±SD)	57.9 (4.9)	58.7 (4.2)	58.0 (4.5)	58.0 (4.2)	0.46
Weighted LDL-C-GRS (±SD)	60.9 (5.2)	61.6 (4.9)	61.2 (4.7)	61.5 (4.9)	0.60
ST elevation myocardial infarction	20 (55.6)	73 (47.7)	62 (33.0)	39 (55.7)	0.40
Non-ST segment myocardial infarction	4 (11.1)	23 (15.0)	36 (19.2)	8 (11.4)	0.79
Unstable angina pectoris	3 (8.3)	12 (7.8)	16 (8.5)	2 (2.9)	0.47
Stable angina pectoris	9 (25.0)	45 (29.4)	74 (39.4)	21 (30.0)	0.28

BMI, body mass index; CAD-GRS, genetic risk score for coronary artery disease; LDL-C-GRS, genetic risk score for low-density lipoprotein cholesterol.

Familial CAD was reported by 41% of the study population. Patients with familial CAD had non-statistically significantly different mean values of CAD-GRS (unweighted scores 68.4 vs. 68.0, p = 0.10, weighted scores 67.7 vs. 67.5, p = 0.49) and LDL-C-GRS (unweighted scores 58.5 vs. 58.3, p = 0.34, weighted scores 63.3 vs. 61.1, p = 0.41), compared with patients without familial CAD. The correlation coefficients between the unweighted CAD-GRS and unweighted LDL-C-GRS were low (r = 0.04, p = 0.10 for the unweighted scores and r = 0.14, p<0.0001 for the weighted scores).

Adjusted for sex, familial CAD was associated with 3.2 years of earlier presentation of CAD, compared with no familial CAD, **Fig 1**. Similar, for each 1 standard deviation high CAD-GRS or LDL-C-GRS, age at revascularization was lowered by approximately 0.5 years, although estimates did not reach statistical significance for CAD-GRS, **Figs 1 and 2**. There was no evidence of effect modifications of the two GRS by smoking, familial CAD, BMI, or gender (p for interactions >0.10).

### Clinical presentation

Patients with premature CAD presented more often with STEMI and less often with stable angina, compared with older individuals, **Tables 1 and 2**. Mean CAD-GRS and the prevalence of familial CAD tended to be similar or lower in STEMI versus the other presentations, **Table 3**. Mean LDL-C-GRS levels were also comparable between the four CAD presentations.

**Table 3:** Mean values (standard deviations) of CAD-GRS and LDL-C-GRS and proportion with familial CAD according to initial CAD presentation.

	STEMI	NSTEMI	UAP	SAP	P for difference
Unweighted CAD-GRS	67.9 (4.8)	69.0 (4.8)	68.7 (4.9)	68.1 (4.6)	0.015
Weighted CAD-GRS	67.4 (4.4)	68.3 (4.3)	68.0 (4.7)	67.5 (4.3)	0.06
Unweighted LDL-C-GRS	58.4 (4.4)	58.2 (4.5)	58.3 (4.5)	58.6 (4.6)	0.60
Weighted LDL-C-GRS	61.2 (4.9)	60.8 (5.1)	61.2 (5.0)	61.5 (5.2)	0.49
N (%) with familial CAD	254 (32.7)	88 (42.9)	51 (47.7)	268 (52.5)	<0.0001

STEMI, ST segment elevation myocardial infarction; NSTEMI, non-ST segment elevation myocardial infarction; UAP, unstable angina pectoris; SAP, stable angina pectoris; CAD-GRS, genetic risk score of coronary artery disease; LDL-C-GRS, genetic risk score of low-density lipoprotein cholesterol.

## Discussion

In the present work, we investigated the relation between familial CAD and influence of genetic predisposition for CAD and LDL-C with the age at onset of CAD requiring surgical interventions in a clinical cohort of patients referred for angiography and undergoing revascularization as part of usual clinical care. Our principal observations are five-fold. First, we observed that familial CAD was associated with 3 years earlier onset of disease, compared with those without familial CAD. Second, there was no correlation of familial CAD with our genetic risk scores and adjustment for familial CAD did not impact the estimates associated with CAD-GRS / LDL-C-GRS (and vice versa). Third, for each standard deviation higher GRS-CAD, age at onset of disease declined by approximately 0.5 years, but was not statistically significant. Fourth, young patients presented more often with ST elevation myocardial infarction and older patients more often with stable angina. The GRS-CAD values were not very different for these two manifestations of CAD. Fifth, although recognized high cholesterol levels were more common in older vs. younger patients, the genetic burden of SNPs predisposing to high blood LDL-C was higher in young vs. older individuals.

Current common SNPs have been estimated to explain only ~11–13% of the heritability of CAD.[15, 16] In line with this, we observed no correlation of familial CAD with the CAD-GRS. A similar and rather modest correlation between familial CAD and genetic risk has previously been reported in the Framingham Heart Study of people without cardiovascular disease (based on a risk score with fewer SNPs),[17] and such weak relation was recently confirmed in the Malmö Diet and Cancer study cohort.[6] It is possible that familial CAD also (to a large extent) reflects clustering of non-genetic risk factors for CAD in addition to capturing CAD genetic risk. For instance, a suboptimal diet was recently associated with 64% of all cardiometabolic deaths in people aged 25–43 years in the U.S. (based on NHANES data).[18] Dietary patterns cluster within families and may even share a significant genetic component.[19, 20] Similar relations of familial clustering, genetic variability, and adverse cardiovascular influence are apparent for e.g. physical inactivity and smoking.[21, 22] Unfortunately, we did not have data on physical activity levels or dietary patterns in our cohort, which could have been helpful in determining the relative importance of adverse lifestyle for age at onset of CAD and to look for gene-by-environment interactions.

Somewhat counterintuitively, we observed that several of the traditional risk factors for CAD, including diabetes, hypertension, known high cholesterol levels, and levels body mass index were higher in those presenting at older age, compared with young individuals. These risk factors tended therefore to be associated with greater age at onset of CAD. Although the observations may be true (if most of the risk in young individuals is conferred by genetics), the paradox could also be well explained if all individuals had equally adverse risk factors and just some individuals had been diagnosed and treated (since treatment postpone onset of CAD). Yet, it is also well known that body mass index, physical inactivity levels, blood pressure, and blood cholesterol levels increases longitudinally with ageing, making the results quite expected.[23] For the present work, we did not have a control group without CAD, which could have been helpful in looking at the relative importance of risk factors in young vs. old individuals (for the overall risk of developing CAD). The increase in cholesterol levels with advancing age may further pose a challenge in the context of familial hypercholesterolemia and many young individuals may be underdiagnosed due to their relatively lower LDL-C concentrations.[24, 25] Our data could be in agreement hereof, where genetic variants predisposing to high LDL-C were higher in young than old individuals, despite that significantly fewer young individuals were diagnosed with high blood cholesterol levels.

Although we observed that those with higher CAD-GRS and LDL-C-GRS presented at younger age, the absolute difference in years were modest (and statistically non-significant for CAD-GRS), suggesting that even if GRS may be of some use in risk stratification in a primary preventive setting (as suggested by prior studies),[6, 7, 26] it may not be a good predictor of age at onset of disease. In accordance with previous studies, we observed that most young individuals presenting with CAD were smokers, which may comprise one of the most important risk factor for early-onset CAD.[7, 27]

Interestingly, women presenting at a very young age had a lower burden of genetic variants associated with the risk of CAD and LDL-C levels than those presenting in mid-life, suggesting that other mechanisms may underlie the development of CAD requiring surgical interventions in very young female individuals. Indeed, normal arteries, coronary artery thrombus, dissection, vasculitis, and coronary anomaly are collectively more common causes than atherosclerotic disease in young women presenting with suspected CAD.[28]

### Strengths and limitations

The major strength of our study included the rather large and clinically representative sample of patients undergoing first-time coronary artery revascularization. Data were, however, collected in a clinical setting and some of it was based on a self-reported history, including the definition of familial CAD, diabetes, and known high cholesterol levels. This may have led to underreporting (potentially due to underdiagnosing) of various disorders. Especially young individuals may not have been adequately screened for risk factors, including diabetes, hypertension, and high blood cholesterol levels prior to presenting with CAD in the coronary artery unit, which may have influenced our observations. Risk time for family members may also have been lower in young patients, which could have contributed to the observed lower prevalence of familial CAD in STEMI vs. the other presentations. It can also not be excluded that some of the young individuals with a high burden of CAD related genes might have died from sudden cardiac death before reaching the catheter room. We did not have data on the indication for revascularization in all individuals. Apart from symptoms of angina or myocardial infarction, some people may have been revascularized because of systolic heart failure (a CAD-GRS is indeed also predictive of onset of systolic heart failure in the community),[29] or ventricular tachycardia. The present study sample was of white European ancestry and it is not known if similar observations would be seen for other populations (e.g. Blacks or Hispanic). Finally, it is likely that our study was statistically underpowered to detect smaller differences in associations of various factors and age at onset of CAD, as well as interactions. With a larger sample, the association between CAD-GRS and age at onset might have been significant and conclusions such that only GRS-LDL-C (in contrast to GRS-CAD) was associated with age at onset should not be drawn. In this context, it should also be noted that although our GRS-CAD and GRS-LDL-C were only weakly correlated, many of the LDL-C related genes have been implicated in CAD and several variants relating to LDL-C were also included in our GRS of CAD.

### Conclusions and clinical implications

Young individuals presenting with CAD requiring surgical interventions had a higher genetic burden of SNPs relating to LDL-C and CAD (although the latter was statistically non-significant), compared with older individuals with CAD. However, the absolute difference was modest, suggesting that genetic screening can currently not be used as an effective prediction tool of when in life a person will develop CAD. Whether undiscovered genetic variants can still explain a “missing heritability” in early-onset CAD warrants more research.

## Supporting information

S1 File (DOCX)

S1 Table (DOCX)

S2 Table (DOCX)
