# Supplementary material for: Association of genetic variants previously implicated in coronary artery disease with age at onset of coronary artery disease requiring revascularizations
Source: PLoS One. 2019 Feb 6;14(2):e0211690. doi: 10.1371/journal.pone.0211690 (PMC6364925; doi:10.1371/journal.pone.0211690)
Supplement: S1 File — (DOCX) [file pone.0211690.s001.docx]

## S1 file. Genotyping, Imputation and Quality control

Genome-wide genotyping was undertaken on the Illumina’s HiScan system at the Novo Nordisk Foundation Centre for Basic Metabolic Research’s laboratory at Symbion, Copenhagen, Denmark. The standard pipeline in Illumina Genome Studio software was used for the genotype calling. SNPs were updated to build 37 (i.e. hg19). Pre-quality control (QC) pipeline, a total of 5904 individuals and 547644 SNPs were included. After QC, we retained a total of 5128 individuals and 539004 SNPs for further analysis. We applied the following QC parameters: 1) a call-rate below 95% (removed n=46 individuals), 2) extreme positive or negative inbreeding coefficients (removed n=56 individuals), 3) ethnic outliers using Principal Component Analysis (PCA) on ancestral markers (removed n=566 individuals), 4) unknown first degree relative relations found by Identical By Descent (IBD) analysis where only the relative with the highest call-rate for each pedigree-pair was retained (removed n=63 individuals), 5) sample duplicates (excluded n=16 individuals), and 6) sex discrepancy between genotype and phenotype data (removed n=29 individuals). In our QC pipeline, a combination of scripts written in Python and R together with PLINK software were used.

Prior to imputation we prepared a VCF from genotypes from the Illumina Core Exome chip, which were aligned to the forward strand of GRCh37 according to the instructions provided by the Sanger imputation service. We excluded non-autosomal variants, variants with a more than 5% missing calls, variants which had a minor allele frequency of less than 0.05, or which diverged from Hardy-Weinberg equibrilium exact test with a p-value of at most 10e-4, leaving in total 260792 variants genotyped in 5671 individuals. We prephased these using Eagle and imputed using pbwt to the Haplotype Reference Consortium (r1.1) via the Sanger imputation server on January 5, 2017.
