# Supplementary material for: Association of genetic variants previously implicated in coronary artery disease with age at onset of coronary artery disease requiring revascularizations
Source: PLoS One. 2019 Feb 6;14(2):e0211690. doi: 10.1371/journal.pone.0211690 (PMC6364925; doi:10.1371/journal.pone.0211690)
Supplement: S1 Table — (DOCX) [file pone.0211690.s002.docx]

## Table S1: Genetic variants comprising polygenic risk score for coronary artery disease

| SNP | Chr | Position | Locus | Effect Allele | Other Allele | Reported EAF | COGEN EAF |
| --- | --- | --- | --- | --- | --- | --- | --- |
| rs11206510 | 1 | [55496039](http://genome.ucsc.edu/cgi-bin/hgTracks?db=hg19&position=chr1:55495988-55496088) | *PCSK9* | T | C | 0.84 | 0.82 |
| rs17114036 | 1 | [56962821](http://genome.ucsc.edu/cgi-bin/hgTracks?db=hg19&position=chr1:56962770-56962870) | *PPAP2B* | A | G | 0.91 | 0.93 |
| rs602633 | 1 | [109821511](http://genome.ucsc.edu/cgi-bin/hgTracks?db=hg19&position=chr1:109821460-109821560) | *SORT1* | C | A | 0.77 | 0.78 |
| rs4845625 | 1 | [154422067](http://genome.ucsc.edu/cgi-bin/hgTracks?db=hg19&position=chr1:154422016-154422116) | *IL6R* | T | C | 0.47 | 0.44 |
| rs17464857 | 1 | [222762709](http://genome.ucsc.edu/cgi-bin/hgTracks?db=hg19&position=chr1:222762658-222762758) | *MIA3* | T | G | 0.18 | 0.14 |
| rs515135 | 2 | [21286057](http://genome.ucsc.edu/cgi-bin/hgTracks?db=hg19&position=chr2:21286006-21286106) | *APOB* | G | A | 0.83 | 0.85 |
| rs6544713 | 2 | [44073881](http://genome.ucsc.edu/cgi-bin/hgTracks?db=hg19&position=chr2:44073830-44073930) | *ABCG5-ABCG8* | T | C | 0.3 | 0.32 |
| rs1561198 | 2 | [85809989](http://genome.ucsc.edu/cgi-bin/hgTracks?db=hg19&position=chr2:85809938-85810038) | *VAMP5-VAMP8-GGCX* | A | G | 0.45 | 0.47 |
| rs2252641 | 2 | [145801461](http://genome.ucsc.edu/cgi-bin/hgTracks?db=hg19&position=chr2:145801410-145801510) | *ZEB2* | G | A | 0.46 | 0.46 |
| rs6725887 | 2 | [203745885](http://genome.ucsc.edu/cgi-bin/hgTracks?db=hg19&position=chr2:203745834-203745934) | *WDR12* | C | T | 0.11 | 0.14 |
| rs1801251 | 2 | [233633460](http://genome.ucsc.edu/cgi-bin/hgTracks?db=hg19&position=chr2:233633409-233633509) | KCNJ13-GIGYF2 | A | G | 0.35 | 0.34 |
| rs9818870 | 3 | [138122122](http://genome.ucsc.edu/cgi-bin/hgTracks?db=hg19&position=chr3:138122071-138122171) | *MRAS* | T | C | 0.14 | 0.18 |
| rs17087335 | 4 | [57838583](http://genome.ucsc.edu/cgi-bin/hgTracks?db=hg19&position=chr4:57838532-57838632) | *REST-NOA1* | T | G | 0.21 | 0.20 |
| rs1878406 | 4 | [148393664](http://genome.ucsc.edu/cgi-bin/hgTracks?db=hg19&position=chr4:148393613-148393713) | *EDNRA* | T | C | 0.15 | 0.15 |
| rs7692387 | 4 | [156635309](http://genome.ucsc.edu/cgi-bin/hgTracks?db=hg19&position=chr4:156635258-156635358) | *GUCY1A3* | G | A | 0.81 | 0.81 |
| rs273909 | 5 | [131667353](http://genome.ucsc.edu/cgi-bin/hgTracks?db=hg19&position=chr5:131667302-131667402) | *SLC22A4-SLC22A5* | C | T | 0.14 | 0.12 |
| rs9369640 | 6 | [12901441](http://genome.ucsc.edu/cgi-bin/hgTracks?db=hg19&position=chr6:12901390-12901490) | *PHACTR1* | A | C | 0.65 | 0.66 |
| rs3130683 | 6 | 31888367 | C2 | T | C | 0.86 | 0.86 |
| rs17609940 | 6 | [35034800](http://genome.ucsc.edu/cgi-bin/hgTracks?db=hg19&position=chr6:35034749-35034849) | *ANKS1A* | G | C | 0.81 | 0.78 |
| rs10947789 | 6 | [39174922](http://genome.ucsc.edu/cgi-bin/hgTracks?db=hg19&position=chr6:39174871-39174971) | *KCNK5* | T | C | 0.76 | 0.77 |
| rs12190287 | 6 | [134214525](http://genome.ucsc.edu/cgi-bin/hgTracks?db=hg19&position=chr6:134214474-134214574) | *TCF21* | C | G | 0.59 | 0.65 |
| rs2048327 | 6 | [160863532](http://genome.ucsc.edu/cgi-bin/hgTracks?db=hg19&position=chr6:160863481-160863581) | LPA | G | A | 0.35 | 0.41 |
| rs3798220 | 6 | [160961137](http://genome.ucsc.edu/cgi-bin/hgTracks?db=hg19&position=chr6:160961086-160961186) | *LPA* | C | T | 0.01 | 0.02 |
| rs4252120 | 6 | [161143608](http://genome.ucsc.edu/cgi-bin/hgTracks?db=hg19&position=chr6:161143557-161143657) | *PLG* | T | C | 0.73 | 0.71 |
| rs2023938 | 7 | [19036775](http://genome.ucsc.edu/cgi-bin/hgTracks?db=hg19&position=chr7:19036724-19036824) | *HDAC9* | G | A | 0.1 | 0.11 |
| rs12539895 | 7 | [107091849](http://genome.ucsc.edu/cgi-bin/hgTracks?db=hg19&position=chr7:107091798-107091898) | 7q22 | A | C | 0.19 | 0.22 |
| rs11556924 | 7 | [129663496](http://genome.ucsc.edu/cgi-bin/hgTracks?db=hg19&position=chr7:129663445-129663545) | *ZC3HC1* | C | T | 0.65 | 0.63 |
| rs3918226 | 7 | [150690176](http://genome.ucsc.edu/cgi-bin/hgTracks?db=hg19&position=chr7:150690125-150690225) | *NOS3* | T | C | 0.06 | 0.09 |
| rs264* | 8 | [9813180](http://genome.ucsc.edu/cgi-bin/hgTracks?db=hg19&position=chr8:19813129-19813229) | *LPL* | G | A | 0.86 | 0.87 |
| rs2954029 | 8 | [126490972](http://genome.ucsc.edu/cgi-bin/hgTracks?db=hg19&position=chr8:126490921-126491021) | *TRIB1* | A | T | 0.55 | 0.54 |
| rs3217992 | 9 | [22003223](http://genome.ucsc.edu/cgi-bin/hgTracks?db=hg19&position=chr9:22003172-22003272) | *CDKN2BAS1* | A | G | 0.38 | 0.38 |
| rs1333049 | 9 | [22125503](http://genome.ucsc.edu/cgi-bin/hgTracks?db=hg19&position=chr9:22125452-22125552) | *CDKN2BAS1* | C | G | 0.47 | 0.50 |
| rs111245230 | 9 | 113169775 | *SVEP1* | C | T | 0.036 | 0.036 |
| rs579459 | 9 | [136154168](http://genome.ucsc.edu/cgi-bin/hgTracks?db=hg19&position=chr9:136154117-136154217) | *ABO* | C | T | 0.21 | 0.22 |
| rs2505083 | 10 | [30335122](http://genome.ucsc.edu/cgi-bin/hgTracks?db=hg19&position=chr10:30335071-30335171) | *KIAA1462* | C | T | 0.42 | 0.43 |
| rs2047009 | 10 | [44539913](http://genome.ucsc.edu/cgi-bin/hgTracks?db=hg19&position=chr10:44539862-44539962) | *CXCL12* | C | A | 0.48 | 0.47 |
| rs501120 | 10 | 44753867 | *CXCL12* | A | G | 0.83 | 0.88 |
| rs11203042 | 10 | [90989109](http://genome.ucsc.edu/cgi-bin/hgTracks?db=hg19&position=chr10:90989058-90989158) | *LIPA* | T | C | 0.44 | 0.43 |
| rs2246833 | 10 | [91005854](http://genome.ucsc.edu/cgi-bin/hgTracks?db=hg19&position=chr10:91005803-91005903) | *LIPA* | T | C | 0.38 | 0.34 |
| rs12413409 | 10 | [104719096](http://genome.ucsc.edu/cgi-bin/hgTracks?db=hg19&position=chr10:104719045-104719145) | *CYP17A1-CNNM2-NT5C2* | G | A | 0.89 | 0.91 |
| rs10840293 | 11 | [9751196](http://genome.ucsc.edu/cgi-bin/hgTracks?db=hg19&position=chr11:9751145-9751245) | *SWAP70* | A | G | 0.55 | 0.58 |
| rs11042937 | 11 | 10745394 | MRVI1-CTR9 | T | G | 0.49 | 0.47 |
| rs974819 | 11 | 103660567 | *PDGFD* | A | G | 0.29 | 0.29 |
| rs9326246 | 11 | [116611733](http://genome.ucsc.edu/cgi-bin/hgTracks?db=hg19&position=chr11:116611682-116611782) | *ZNF259-APOA5-APOA1* | C | G | 0.90 | 0.93 |
| rs11172113 | 12 | [57527283](http://genome.ucsc.edu/cgi-bin/hgTracks?db=hg19&position=chr12:57527232-57527332) | LRP1 | C | T | 0.41 | 0.43 |
| rs3184504 | 12 | 111884608 | *SH2B3* | T | C | 0.4 | 0.46 |
| rs11057830 | 12 | [125307053](http://genome.ucsc.edu/cgi-bin/hgTracks?db=hg19&position=chr12:125307002-125307102) | SCARB1 | A | G | 0.15 | 0.14 |
| rs9319428 | 13 | [28973621](http://genome.ucsc.edu/cgi-bin/hgTracks?db=hg19&position=chr13:28973570-28973670) | *FLT1* | A | G | 0.32 | 0.31 |
| rs4773144 | 13 | [110960712](http://genome.ucsc.edu/cgi-bin/hgTracks?db=hg19&position=chr13:110960661-110960761) | *COL4A1-COL4A2* | G | A | 0.42 | 0.42 |
| rs9515203 | 13 | [111049623](http://genome.ucsc.edu/cgi-bin/hgTracks?db=hg19&position=chr13:111049572-111049672) | *COL4A1-COL4A2* | T | C | 0.74 | 0.73 |
| rs2895811 | 14 | [100133942](http://genome.ucsc.edu/cgi-bin/hgTracks?db=hg19&position=chr14:100133891-100133991) | *HHIPL1* | C | T | 0.42 | 0.43 |
| rs56062135 | 15 | [67455630](http://genome.ucsc.edu/cgi-bin/hgTracks?db=hg19&position=chr15:67455579-67455679) | *SMAD3* | C | T | 0.79 | 0.77 |
| rs7173743 | 15 | [79141784](http://genome.ucsc.edu/cgi-bin/hgTracks?db=hg19&position=chr15:79141733-79141833) | *ADAMTS7* | T | C | 0.58 | 0.58 |
| rs8042271 | 15 | [89574218](http://genome.ucsc.edu/cgi-bin/hgTracks?db=hg19&position=chr15:89574167-89574267) | *MFGE8-ABHD2* | G | A | 0.9 | 0.95 |
| rs17514846 | 15 | [91416550](http://genome.ucsc.edu/cgi-bin/hgTracks?db=hg19&position=chr15:91416499-91416599) | *FURIN-FES* | A | C | 0.44 | 0.47 |
| rs1800775 | 16 | [56995236](http://genome.ucsc.edu/cgi-bin/hgTracks?db=hg19&position=chr16:56995185-56995285) | CETP | C | A | 0.51 | 0.47 |
| rs2281727 | 17 | [2117945](http://genome.ucsc.edu/cgi-bin/hgTracks?db=hg19&position=chr17:2117894-2117994) | *SMG6* | C | T | 0.36 | 0.37 |
| rs12936587 | 17 | [17543722](http://genome.ucsc.edu/cgi-bin/hgTracks?db=hg19&position=chr17:17543671-17543771) | *RAI1-PEMT-RASD1* | G | A | 0.59 | 0.54 |
| rs15563 | 17 | [47005193](http://genome.ucsc.edu/cgi-bin/hgTracks?db=hg19&position=chr17:47005142-47005242) | *UBE2Z* | C | T | 0.52 | 0.54 |
| rs7212798* | 17 | [59013488](http://genome.ucsc.edu/cgi-bin/hgTracks?db=hg19&position=chr17:59013437-59013537) | *BCAS3* | C | T | 0.15 | 0.15 |
| rs663129 | 18 | [57838401](http://genome.ucsc.edu/cgi-bin/hgTracks?db=hg19&position=chr18:57838350-57838450) | *PMAIP1-MC4R* | A | G | 0.26 | 0.25 |
| rs116843064 | 19 | 8429323 | *ANGPTL4* | A | G | 0.02 | 0.02 |
| rs1122608 | 19 | [11163601](http://genome.ucsc.edu/cgi-bin/hgTracks?db=hg19&position=chr19:11163550-11163650) | *LDLR* | G | T | 0.76 | 0.77 |
| rs2075650 | 19 | [45395619](http://genome.ucsc.edu/cgi-bin/hgTracks?db=hg19&position=chr19:45395568-45395668) | *APOE-APOC1* | G | A | 0.14 | 0.18 |
| rs445925 | 19 | [45415640](http://genome.ucsc.edu/cgi-bin/hgTracks?db=hg19&position=chr19:45415589-45415689) | *APOE-APOC1* | C | T | 0.9 | 0.91 |
| rs9982601 | 21 | [35599128](http://genome.ucsc.edu/cgi-bin/hgTracks?db=hg19&position=chr21:35599077-35599177) | *KCNE2* | T | C | 0.13 | 0.14 |
| rs180803* | 22 | [24658858](http://genome.ucsc.edu/cgi-bin/hgTracks?db=hg19&position=chr22:24658807-24658907) | *POM121L9P-ADORA2A* | G | T | 0.97 | 0.99 |

Footnote: Overall, 67 independent genomic variants associated with coronary heart disease at stringent statistical thresholds have been found.^1-4^ In construction of the polygenic risk scores, we included directly genotyped and imputed proxy variants (r^2^ > 0.8). *Instead of rs264 we used rs256 as a proxy variant (r^2^=1); instead of rs180803 we used rs5760293 as a proxy variant (r^2^=1); instead of rs7212798 we used rs747895 as a proxy variant (r^2^=1). r^2^ for proxy variants identified from <https://analysistools.nci.nih.gov/LDlink/> using a central European reference population.

**References:**

1. Brewer F, Bhattacharyya L, Brown RD, 3rd, Koenig SH. Interactions of concanavalin A with a trimannosyl oligosaccharide fragment of complex and high mannose type glycopeptides. *Biochem Biophys Res Commun.* 1985;127(3):1066-1071.

2. Myocardial Infarction G, Investigators CAEC, Stitziel NO, et al. Coding Variation in ANGPTL4, LPL, and SVEP1 and the Risk of Coronary Disease. *N Engl J Med.* 2016;374(12):1134-1144.

3. Webb TR, Erdmann J, Stirrups KE, et al. Systematic Evaluation of Pleiotropy Identifies 6 Further Loci Associated With Coronary Artery Disease. *J Am Coll Cardiol.* 2017;69(7):823-836.

4. Nikpay M, Goel A, Won HH, et al. A comprehensive 1,000 Genomes-based genome-wide association meta-analysis of coronary artery disease. *Nat Genet.* 2015;47(10):1121-1130.
