# Supplementary material for: Association of genetic variants previously implicated in coronary artery disease with age at onset of coronary artery disease requiring revascularizations
Source: PLoS One. 2019 Feb 6;14(2):e0211690. doi: 10.1371/journal.pone.0211690 (PMC6364925; doi:10.1371/journal.pone.0211690)
Supplement: S2 Table — (DOCX) [file pone.0211690.s003.docx]

## Table S2: Genetic variants comprising polygenic risk score for low-density-lipoprotein cholesterol (LDL-C)

| SNP | Chr | Position | Locus | Effect Allele | Other Allele | Reported EAF | COGEN EAF |
| --- | --- | --- | --- | --- | --- | --- | --- |
| rs12027135 | 1 | 25775733 | *LDLRAP1* | A | T | 0.46 | 0.44 |
| rs12748152 | 1 | 27138393 | *PIGV-NR0B2* | T | C | 0.09 | 0.09 |
| rs2479409 | 1 | 55504650 | *PCSK9* | G | A | 0.32 | 0.35 |
| rs2131925 | 1 | 63025942 | *ANGPTL3* | G | T | 0.34 | 0.34 |
| rs629301 | 1 | 109818306 | *SORT1* | G | T | 0.24 | 0.22 |
| rs2642442 | 1 | 220973563 | *MOSC1* | C | T | 0.33 | 0.28 |
| rs514230 | 1 | 234858597 | *IRF2BP2* | A | T | 0.48 | 0.47 |
| rs267733 | 1 | [150958836](https://www.ncbi.nlm.nih.gov/variation/view/?q=rs267733&filters=source:dbsnp&assm=GCF_000001405.25) | *ANXA9-CERS2* | G | A | 0.16 | 0.15 |
| rs1367117 | 2 | [21263900](https://www.ncbi.nlm.nih.gov/variation/view/?q=rs1367117&filters=source:dbsnp&assm=GCF_000001405.25) | *APOB* | A | G | 0.32 | 0.33 |
| rs4299376 | 2 | 44072576 | *ABCG5/8* | G | T | 0.31 | 0.32 |
| rs2710642 | 2 | 63149557 | *EHBP1* | G | A | 0.35 | 0.32 |
| rs10490626 | 2 | 118835841 | *INSIG2* | A | G | 0.08 | 0.06 |
| rs2030746 | 2 | 121309488 | *LOC84931* | T | C | 0.4 | 0.42 |
| rs1250229 | 2 | 216304384 | *FN1* | T | C | 0.27 | 0.26 |
| rs11563251 | 2 | 234679384 | *UGT1A1* | T | C | 0.12 | 0.10 |
| rs7640978 | 3 | 32533010 | *CMTM6* | T | C | 0.09 | 0.08 |
| rs17404153 | 3 | 132163200 | *ACAD11* | T | G | 0.14 | 0.10 |
| rs6818397 | 4 | 3434885 | *LRPAP1* | T | G | 0.37 | 0.38 |
| rs12916 | 5 | 74656539 | *HMGCR* | C | T | 0.4 | 0.41 |
| rs4530754 | 5 | 122855416 | *CSNK1G3* | G | A | 0.46 | 0.46 |
| rs6882076 | 5 | 156390297 | *TIMD4* | T | C | 0.36 | 0.36 |
| rs3757354 | 6 | 16127407 | *MYLIP* | T | C | 0.24 | 0.25 |
| rs1800562 | 6 | [26093141](https://www.ncbi.nlm.nih.gov/variation/view/?q=rs1800562&filters=source:dbsnp&assm=GCF_000001405.25) | *HFE* | A | G | 0.07 | 0.05 |
| rs3177928 | 6 | 32412435 | *HLA* | A | G | 0.17 | 0.15 |
| rs9488822 | 6 | 116312893 | *FRK* | T | A | 0.36 | 0.30 |
| rs1564348 | 6 | 160578860 | *LPA* | C | T | 0.18 | 0.17 |
| rs12670798 | 7 | 21607352 | *DNAH11* | C | T | 0.25 | 0.24 |
| rs4722551 | 7 | 25991826 | *MIR148A* | C | T | 0.2 | 0.15 |
| rs2072183 | 7 | 44579180 | *NPC1L1* | C | G | 0.29 | 0.22 |
| rs9987289 | 8 | 9183358 | *PPP1R3B* | A | G | 0.1 | 0.10 |
| rs10102164 | 8 | 55421614 | *SOX17* | A | G | 0.21 | 0.22 |
| rs2081687 | 8 | 59388565 | *CYP7A1* | T | C | 0.36 | 0.33 |
| rs2954029 | 8 | 126490972 | *TRIB1* | T | A | 0.47 | 0.46 |
| rs11136341 | 8 | 145043543 | *PLEC1* | G | A | 0.4 | 0.36 |
| rs3780181 | 9 | 2640759 | *VLDLR* | G | A | 0.08 | 0.06 |
| rs9411489* | 9 | [136155000](https://www.ncbi.nlm.nih.gov/variation/view/?q=rs635634&filters=source:dbsnp&assm=GCF_000001405.25) | *ABO* | T | C | 0.21 | 0.21 |
| rs2255141 | 10 | 113933886 | *GPAM* | A | G | 0.3 | 0.28 |
| rs174546 | 11 | 61569830 | *FADS1-2-3* | T | C | 0.36 | 0.34 |
| rs964184 | 11 | 116648917 | *APOA1* | C | G | 0.84 | 0.85 |
| rs11220462 | 11 | 126243952 | *ST3GAL4* | A | G | 0.14 | 0.13 |
| rs11065987 | 12 | 112072424 | *BRAP* | G | A | 0.41 | 0.47 |
| rs1169288* | 12 | [121416650](https://www.ncbi.nlm.nih.gov/variation/view/?q=rs1169288&filters=source:dbsnp&assm=GCF_000001405.25) | *HNF1A* | C | A | 0.34 | 0.34 |
| rs4942486 | 13 | 32953388 | *BRCA2* | T | C | 0.48 | 0.49 |
| rs8017377 | 14 | [24883887](https://www.ncbi.nlm.nih.gov/variation/view/?q=rs8017377&filters=source:dbsnp&assm=GCF_000001405.25) | *NYNRIN* | A | G | 0.46 | 0.47 |
| rs3764261 | 16 | 56993324 | *CETP* | A | C | 0.32 | 0.31 |
| rs2000999 | 16 | 72108093 | *HPR* | A | G | 0.2 | 0.22 |
| rs314253 | 17 | 7091650 | *DLG4* | C | T | 0.37 | 0.38 |
| rs7206971 | 17 | 45425115 | *OSBPL7* | A | G | 0.49 | 0.48 |
| rs1801689* | 17 | [64210580](https://www.ncbi.nlm.nih.gov/variation/view/?q=rs1801689&filters=source:dbsnp&assm=GCF_000001405.25) | *APOH-PRXCA* | C | A | 0.04 | 0.02 |
| rs6511720 | 19 | 11202306 | *LDLR* | T | G | 0.12 | 0.09 |
| rs10401969 | 19 | 19407718 | *CILP2* | C | T | 0.09 | 0.08 |
| rs4420638 | 19 | 45422946 | *APOE* | G | A | 0.19 | 0.21 |
| rs364585 | 20 | 12962718 | *SPTLC3* | A | G | 0.38 | 0.39 |
| rs2328223 | 20 | 17845921 | *SNX5* | C | A | 0.21 | 0.16 |
| rs2902940 | 20 | 39091487 | *MAFB* | G | A | 0.3 | 0.28 |
| rs6029526 | 20 | 39672618 | *TOP1* | A | T | 0.47 | 0.48 |
| rs5763662 | 22 | 30378703 | *MTMR3* | T | C | 0.04 | 0.02 |
| rs4253776 | 22 | 46629479 | *PPARA* | G | A | 0.13 | 0.10 |

Overall, 58 independent genomic variants associated with at stringent statistical thresholds have been found. In construction of the polygenic risk scores, we included directly genotyped and imputed proxy variants (r^2^ > 0.8).^5^ *Instead of rs1801689 we used [rs149394327](http://www.ncbi.nlm.nih.gov/projects/SNP/snp_ref.cgi?rs=149394327) as a proxy variant (r^2^=1); instead of rs1169288 we used [rs2244608](http://www.ncbi.nlm.nih.gov/projects/SNP/snp_ref.cgi?rs=2244608) as a proxy variant (r^2^= 0.9771); instead of rs635634 we used [rs532436](http://www.ncbi.nlm.nih.gov/projects/SNP/snp_ref.cgi?rs=532436) as a proxy variant (r^2^= 0.9689). r^2^ for proxy variants identified from <https://analysistools.nci.nih.gov/LDlink/> using a central European reference population.

5. Global Lipids Genetics C, Willer CJ, Schmidt EM, et al. Discovery and refinement of loci associated with lipid levels. *Nat Genet.* 2013;45(11):1274-1283.
